# Observations on the Use of Cold Water in Typhus

**Published:** 1802-08-01

**Authors:** 

**Affiliations:** Surgeon, of Oxford Street


*Obſervations on the Uſe of cold Water in Typhus; communicated by Mr.* BLEGBOROUGH, *Surgeon, of Oxford Street.*

SHOULD I advance nothing *new* on the preſent occaſion, at leaſt let me be allowed the merit of intending to bring, ſub judice, and fix attention to, a ſubject, than which none calls louder for a cool and diſpaſſionate inveſtigation, and for which no place is more proper than your pages, from whence ſuffering humanity has already reaped ſo many advantages.

Need I mention more urgent reaſons for the neceſſity of an inveſtigation of the treatment of Typhus Fever, than that it has ſo lately removed a Letſom, a Murray, a Garnett, and others, bright ornaments of Humanity as well as of a Profeſſion, which has lately claimed its proper rank in ſociety, and to which it is my greateſt glory to belong.

I ſhall make no other apology for intruding myſelf upon the notice of your Readers, than that, unſhackled by prejudice, I have attended minutely to what of the diſeaſe occurred to me in a practice of the laſt ten years, ſo active as to preclude me the privilege of examining the opinions of others, and from having with ſome of my own family paſſed the fiery ordeal myſelf.

From the commencement of Typhus we have heat (from increaſed circulation, or whatever other cauſe is at preſent immaterial, as the cauſes of the diſeaſe are not intended to have any thing to do with the preſent inveſtigation); we have heat then, and that not as Dr. Cullen ſays, parum but plurimum auctus, and the patient ſuffers in a ratio exactly commenſurate to the degree and duration of that increaſed heat. By it are produced thoſe conſequences with which we are all but too well acquainted. By heat the ſecretions are locked up, the gently promoting which, has ever ſeemed to me a circumſtance of the firſt importance in the cure of Typhus. The ſecretions are moſt eaſily kept up, the functions, particularly of the ſtomach, more duly performed, and the vigour of the whole ſyſtem longeſt ſuſtained, while the body is kept cool. Heat produces anxiety of mind and reſtleſſneſs of body, inducing too high a ſtate of excitement to be long ſuſtained with impunity. The functions of the ſtomach then being impaired, the ordinary practice of adminiſtering bark, wine, brandy, and opium, tends, inſtead of diminiſhing, to increaſe that heat upon which the miſchief depends, and which, if not reſtrained, ſoon hurries matters from bad to worſe. Theſe, in my opinion, at leaſt now, no longer act upon the ſtomach, according to calculations drawn from their effects on it in its healthy ftate. Their adminiftration feems to me like heaping Pelion on Offa on the patient. They furely are not the propereft things to pafs the ftomach in fuch a ftate, but ought to give place to lac amygdalæ, paftry-cook-whey, or even common water. It may not be amifs here to remark, that nothing ought to be preffed on the patient, or put into his ftomach contrary to his wifh; but that his own feelings fhould, as nearly as poffible, be confulted in this affair. The ventilation of the room, and the moderating the warmth of the bed-cloaths, are things alfo of the utmoft importance. Were it poffible for me to forget the effects which even a fmall blifter had upon myfelf, I might perhaps be induced to think fomewhat lefs unfavourably of it in the cafes of others. I may be miftaken in all this; but if I am, I cannot make out for what purpofes my fenfes were given me. No man of common fenfe will, I am fure, find any thing unreafonable in what I have advanced, which would not even be improbable, did not every day's experience prove that it is really true. Do the Phyficians of London, then, doubt the truth of Dr. Currie's remarks on the fubject? or what other caufe withholds their fanction from a treatment which reafon fuggefts and experience approves?

I have been told that no one wifhes to be the firft to hazard a reputation, with difficulty obtained, on a novel fyftem, though under the moft thorough conviction of its propriety. Do phyficians, indeed, find the iron chain of vulgar prejudice fo very ftrong? No! this I will not believe, while I know a Saunders, a Bailey, yourfelves, gentlemen, and others, whofe liberality of fentiment, independence of fpirit, and goodnefs of heart are only equalled by their profeffional fkill. The little reputation I have, has been, and were it equal to that of your whole College, fhould all be placed on this fingle dye, fo convinced am I that it would not be fullied by the rifk.

We are then to look to fome other caufe why the plan is not more generally adopted; and the moft likely one perhaps is, that eminent men conftantly engaged in an active and arduous purfuit, have not yet beftowed on it that degree of attention, which the thing feems really to deferve. I know it has been faid that there have been inftances in which fudden affufion has deprived the patient of life altogether in an inftant. I don't know that it is true, having never feen nor heard of a well authenticated Cafe of it. Admitted, however, to be true, I do not think it fo much the fault of the remedy as the improper application of it. The beft remedies of every defcription, when mifapplied, are capable of producing the greateft mifchief; and the whole art of medicine confifts in properly apportioning apportioning the degree of remedy to the degree of difeafe, which, more than any other thing, diftinguifhes the phyfician from the quack. In avoiding Scylla, are we of neceffity to get into Charybdis ?

To lay down fpecific rules by which I have regulated the application of cold, would not be eafy ; as circumftances are not exactly alike in any two cafes, and as every patient's conftitution and habits form an exception from a general rule.

After, in fome cafes only, premifing an emetic, I have in general found little further neceffary than fimply to bathe the whole of the body with cold water, with or without a fmall quantity of vinegar, by means of a napkin or fponge, three or four times a day.

I have feldom given any thing by way of medicine, except a draught three times a day of lac amygdalæ, with fometimes three grains of nitre in each, and that more as a placebo than with any other intention. But I have always made a great object of attending to the ftate of the bowels, and have generally had an enema, more or lefs aperient, according to circumftances, adminiftered every, or every other, evening, till the fourteenth ; at which period, under this treatment, the patient feldom, if ever, fails to find himfelf completely free from the diforder.

In fome bad cafes, however, where matters have been fuffered to run into extremes from inattention in the earlier ftages of the difeafe, I have (in order to get the heat more quickly out of the habit), been under the neceffity of employing affufion ; yet, I have always thought it right to ufe the bathing firft, well aware that Nature hates fudden tranfitions, not lefs than a *vacuum*.

I was lately called to a patient in an early ftage of the difeafe, and was acting up to the fpirit of the principles laid down in this letter, when an eminent phyfician was called. He very politely faw no reafon to interfere with the treatment for the firft three days, which got us on to about the eighth of the difeafe ; when, concluding that the fever was gone, he directed the cortex, and a fufpenfion of the bathing ; *this*, as I had predicted, reproduced the bad fymptoms, for which I had been originally called. I was then applied to in the moft folemn manner to declare, what I thought was really the ftate of the cafe ? My reply was, that they were already in poffeffion of my opinions. I was made to believe that the vifits of the phyfician were difpenfed with, and knew very well that the original teatment was refumed.

Matters foon got into their proper channels again, and I was not told till the fourteenth day, when I had faid the patient would be be better, that the physician had been attending the whole time; and had declared, that he never saw a patient suffer less from the disease. It may be said, why was the physician called at all?

The novelty of the treatment, and my having said that the patient should confine himself, and use care till the fourteenth day, was, I have no doubt, the cause.

Never having read Dr. Currie's book, I believe I differ from him in opinion, that the disease may be entirely cured by two or three affusions. Though suspended by affusion, as the discharge of gonorrhœa may be by an astringent injection, yet I have no doubt that the disease will certainly become active again, and the heat accumulate as in gonorrhœa, if the remedies, equally specific in each, be for a short time suspended. In my opinion, if ever typhus be protracted beyond the fourteenth day, if much head-ach, delirium, putrescency, and all their terrible consequences supervene, it will be always found to be the case, where a different plan to the one here recommended has been pursued. The risk of contagion also, if not entirely removed, is at least rendered infinitely less probable, while the feelings of the patients themselves throughout the whole course of it, leave no doubt of its being the most pleasant one of any before adopted. Another pleasant circumstance I have observed, is that patients treated on the above plan seldom feel much inconvenience afterwards, while those who survive the one in general usage, are often very infirm all the rest of their lives. We have been lately amused with a story, that digitalis was a certain specific in typhus. The result of my observation on this dangerous remedy, for the best of all reasons, extends no further than to one case, in which I was induced to give my attention to its exhibition in the way recommended, viz. a scruple in infusion at four doses in the space of twenty-four hours. The patient died about the ninth day of the disease.

Eheu! Phryges serò sapiebant!

In a conversation with the ingenious but ill-requited Dr. Jenner, at the Royal Institution, the other evening, I think I discovered, that his opinions on this subject were not very different to those here stated, which emboldens me much in thus exhibiting them to the world.

Aware of the hint you gave me respecting the length of my last, I shall content myself at present with having simply started the subject, and shall be happy to see the justice it deserves done to it by others, more equal to the task.

